# Abstract of Proceedings of the Buffalo Medical Association

**Published:** 1869-05

**Authors:** T. M. Johnson

**Affiliations:** Sec’y.


ART. II.—*Abstract of Proceedings of the Buffalo Medical Association.*

TUESDAY EVENING, May 4th, 1869.

Dr. J. F. Miner, President, in the chair. Members present—Drs. Miner, Ring, Rochester, Taylor, Daggett, Strong, Gay and Johnson.

DR. MINER presented the specimen of "neuter gender," reported by Dr. Little at the last meeting, and by careful dissection showed the entire absence of sexual organs. There appeared nothing different from the descriptions by Drs. Miner and Little, published in the transactions for last month. It was the unanimous opinion of those present that it was a very rare, perhaps an unheard of, anomaly in human construction.

DR. MINER also presented an atrophied liver, which he had the day before removed from a patient thirty-eight years old, who, up to within about two years, had enjoyed excellent health. About one year since she came under his observation for the first time, supposing herself to have ovarian tumor. Examination satisfied him that this was an error, and nothing was done until about four months since, when she was tapped for ascites, when about twenty-four quarts of serum were drawn off. This operation was repeated about every three weeks up to the time of death.

When the serum was completely drawn off a small, hard, movable tumor could be distinctly seen and felt a little to the right of the lower border of the sternum. It was obviously a part of, or attached to the liver, believed to be a part of that organ. Upon *post mortem* examination the liver was found atrophied so as to weigh only two pounds. It was roughened, irregular, hardened and deformed, so as to not resemble a liver in any respect; its fibrous sheath thickened and attached to neighboring tissues throughout nearly its entire extent. The *ductus communis choledochus* was obstructed by a biliary concretion, and the gall bladder very fully distended. The spleen was found enlarged and softened.

The appearance of the organ corresponded more with his views of the condition in cirrhosis than any other disease of the liver with which he was acquainted, but he had never seen such a liver as the one shown, and thought it must be a very rare thing for the organ to reach such a degree of deformity and contraction. He had presented it before the Society hoping that some member would be able to give more accurately than himself the pathology of the disease.

Dr. Rochester said that he had examined many livers, but had never seen one like this, and did not know what it was. It certainly was not cirrhosis as usually found; he should call it atrophy with induration, the result possibly of innutrition from disease of the hepatic artery or vena portæ.

Dr. C. C. F. Gay reported the following case of supposed embolism: Mrs. W., aged thirty-two years, the mother of a healthy child three years old. The first labor was protracted and the child was delivered with forceps in the absence of labor pain. This labor was followed for ten days by a fever, when the pulse was a hundred and twenty, with but little variation until convalescence; also copious perspiration. After her recovery she was in good health until ten days before her last confinement, when she rode out on a cold day in an open carriage, and felt and expressed the belief that she had taken a cold that would result in her death. On the following day she had a chill followed by fever, and pain, referred to the right shoulder, and was thereafter unable to lie only upon the left side; change of position to the back or right side caused pain and spasmodic cough. Labor commenced on the evening of the eighteenth of February, progressed favorably, and terminated naturally, and was of four and a half hours' duration. Chloroform was given from the commencement of the labor. There was but little hemorrhage during or after the labor. During labor she could lie upon her back without pain or discomfort, but soon after was obliged to lie upon the left side as before labor, and continued to lie in this position until she died. The pulse during the whole of her sickness until the morning of the ninth day after confinement, one hundred and twenty per minute. Respiration twenty-eight per minute; tongue red, and perspiration profuse. There was no tenderness of the abdomen and no tympanitis; no pain except of the fore finger of the left hand and in one foot. Lacteal secretion was established on the third day and continued with the lochial secretion until death—both scanty. Quite severe chill on the morning of the ninth day after accouchment, after which the pulse gradually increased in frequency until the same night when she died. The voice was full and strong during the whole course of her sickness; mind clear, no delirium. Died suddenly on the evening of the ninth day, after having taken food and conversing as usual.

Dr. Strong reported case of sudden death. A drayman, apparently in good health until the day of his death, got up in the morning not feeling as well as usual, had headache, was about his business all day, partook of his usual supper and felt as well as usual, laid down upon his bed to rest, and twenty minutes later was found by his wife in a kind of convulsion, and died in a short time. Was not satisfied as to cause of death, and could not obtain a *post mortem* examination. Did not ascertain that he had heart disease.

By vote, the Secretary was directed to publish the Constitution and By-Laws.

Dr. Gay moved that Dr. Daggett be appointed a delegate to the State Medical Society of Pennsylvania, during the annual meeting for the present year. Carried.

The following resolutions, introduced by Dr. Miner at a previous meeting, were passed unanimously:

*Resolved,* That the fee for examination for life insurance shall be five dollars when made in the offices of physicians, and two dollars additional when the examination is made outside the office.

*Resolved,* That it shall be regarded as a breach of confidence for druggists to renew prescriptions upon which is written "*not to be renewed,*" or any other sign or device by which it can be known that such renewal is against the wishes of the prescriber.

Adjourned. T. M. Johnson, Sec'y.

---

Hair-bleaching from Neuralgia.—The influence of neuralgia in decolorizing the hair is well known in the case of a patient lately under Dr. Murray's care in the Great Northern Hospital. This woman, who was admitted on account of a fibrous tumor of the uterus, was remarkable in having the internal half of one eyebrow and the corresponding portion of eyelashes perfectly white, she being a brunette. It seems that three or four years ago, having gone to bed well, she was attacked during the night with a very severe spasmodic tic, which lasted only a few minutes; and in the morning the hair was permanently bleached.—*Lancet.*

